# Management of a High-Beta-Human Chorionic Gonadotropin (β-HCG) Ectopic Pregnancy With Methotrexate: A Case Report

**DOI:** 10.7759/cureus.75783

**Published:** 2024-12-16

**Authors:** Athanasios Petroulakis, Bing Bing Tiong

**Affiliations:** 1 Obstetrics and Gynaecology, Nottingham University Hospitals NHS Trust, Nottingham, GBR

**Keywords:** high-dose methotrexate, management of ectopic, pregnancy counseling, tubal ectopic pregnancy, β-hcg

## Abstract

Ectopic pregnancy is a severe complication of early pregnancy that can lead to significant morbidity and mortality. Methotrexate is a standard non-surgical treatment for ectopic pregnancy, especially in cases where beta-human chorionic gonadotropin (β-HCG) levels are low. However, its effectiveness decreases as β-HCG levels rise. We present a case of a 38-year-old nulliparous woman with a high-β-HCG level ectopic pregnancy, who was managed successfully with methotrexate despite initial concerns about potential treatment failure. This case underscores the importance of patient-centered care, thorough counseling, and a multidisciplinary approach in managing complex ectopic pregnancies, particularly when standard guidelines may not fully apply. The patient was closely monitored and achieved a successful outcome without the need for surgery.

## Introduction

Ectopic pregnancy, defined as the implantation of a fertilised egg outside the uterine cavity, occurs in approximately 1-2% of all pregnancies [1] and remains a leading cause of maternal mortality in the first trimester. Early detection and prompt treatment are essential to prevent complications such as tubal rupture, haemorrhage, and, subsequently, hypovolemic shock. Methotrexate, a folate antagonist, has become a well-established medical treatment option for ectopic pregnancy, particularly in cases where β-HCG levels are relatively low and the patient is haemodynamically stable [2]. Its mechanism of action involves the inhibition of DNA synthesis, effectively halting the proliferation of trophoblastic tissue, and is often favoured due to its non-invasive nature and preservation of tubal function. However, when β-HCG levels are markedly elevated, the likelihood of methotrexate failure increases, often making surgical intervention the recommended course of action [3]. Factors such as the size of the ectopic mass, the presence of fetal cardiac activity, and the patient’s overall clinical stability are also critical in determining the appropriateness of methotrexate therapy. In this report, we present a case of a high-β-HCG level ectopic pregnancy managed successfully with methotrexate, illustrating the role of individualised patient care and the potential for non-surgical management in selected cases.

## Case presentation

Situation and background

A 38-year-old nulliparous woman presented to the early pregnancy assessment unit with vaginal bleeding and left iliac fossa pain. Based on the last menstrual period (LMP), the patient was six weeks pregnant. There was no significant past medical history. Regarding the surgical history, the patient had a benign skin cyst removed from the chest and developed a keloid scar. There was no pelvic inflammatory disease (PID) history.

Assessment

**Figure 1:**
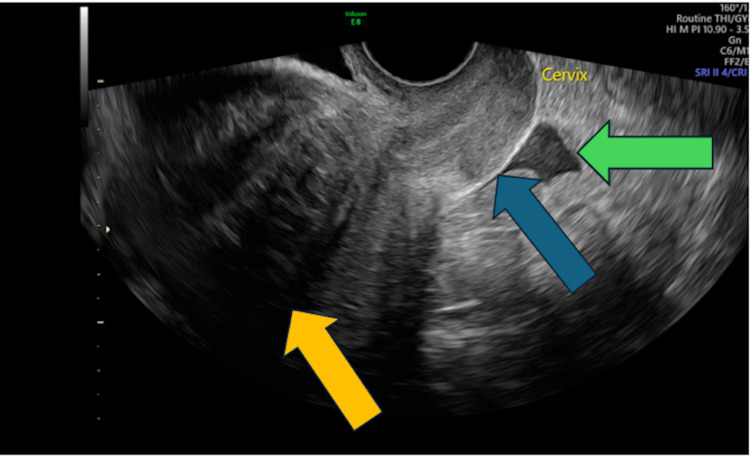
Transvaginal ultrasound: the cervix is seen (blue arrow), with a mild amount of free fluid (green arrow) and a fibroid with shadowing (orange arrow).

**Figure 2:**
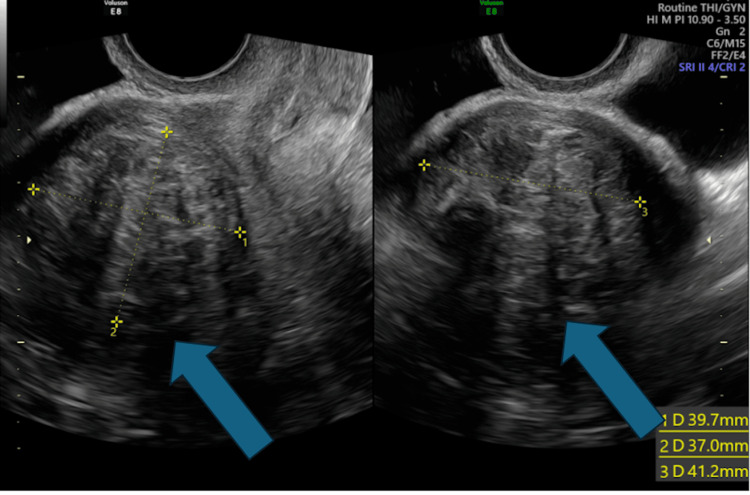
Transvaginal ultrasound: An anterior intramural fibroid is visualised, measuring 39.7 x 37 x 41.2 mm, in different axes as shown by the blue arrows.

**Figure 3:**
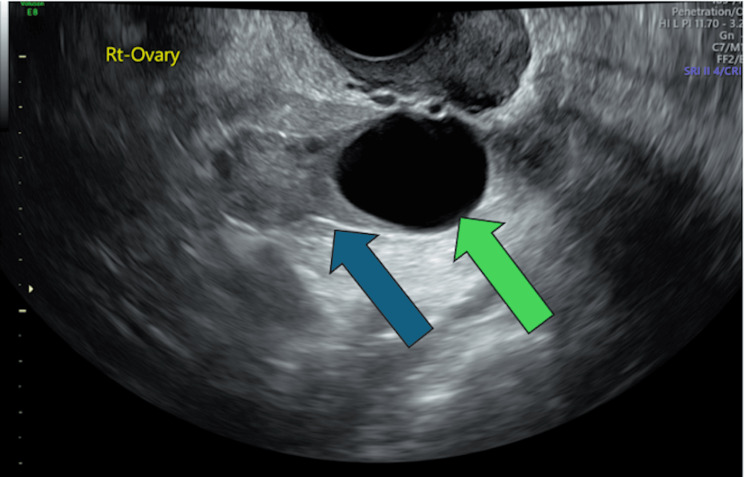
Transvaginal ultrasound: The right ovary (blue arrow) is seen, along with the dominant follicle (green arrow).

**Figure 4:**
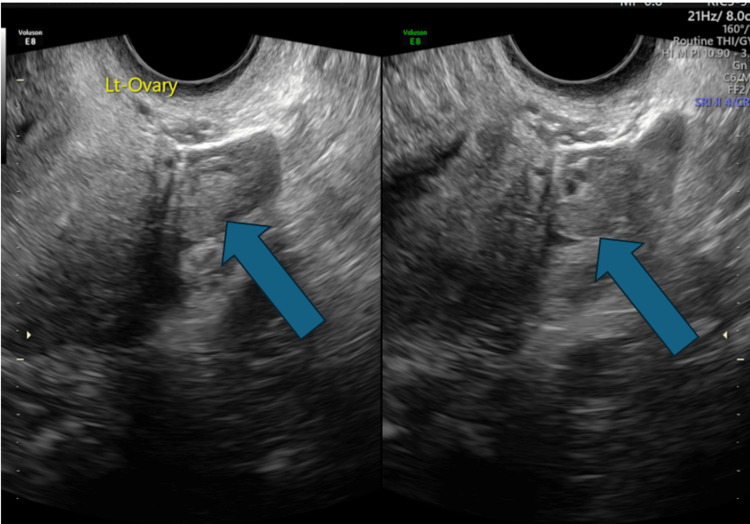
Transvaginal ultrasound: The left ovary (blue arrow) appears normal.

**Figure 5:**
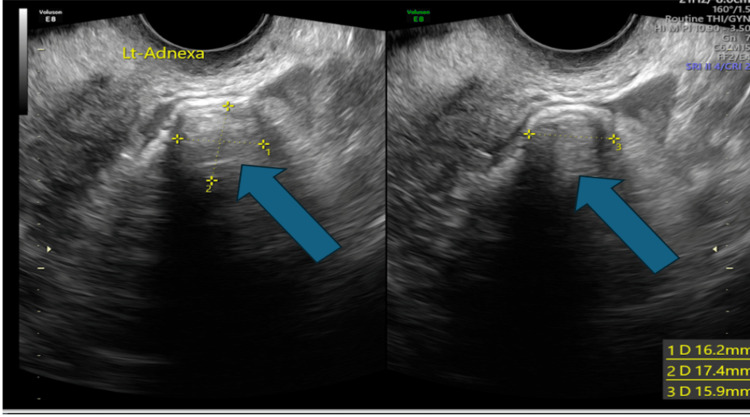
Different axes of the left adnexa. A 16.2 x 17.4 x 17.9 mm mass is visualised in the left adnexa (blue arrow) – possible ectopic pregnancy.

**Figure 6:**
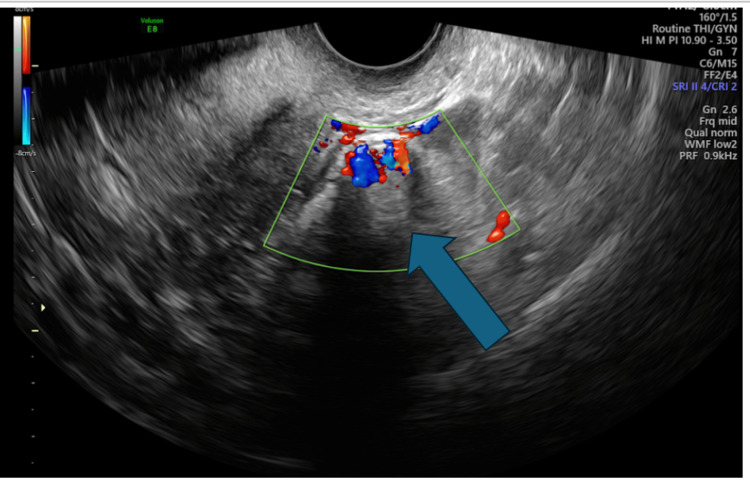
Transvaginal ultrasound (mode Doppler): Vascularity is seen within the possible ectopic pregnancy (blue arrow).

The initial β-HCG level was 2,866 kU/L, which doubled to 4,186 kU/L after 48 hours. An ultrasound scan at that time showed a pregnancy of unknown location (PUL). Three days later, the β-HCG level rose to 6,575 kU/L; a week later, it reached the value of 20,130 kU/L. A repeat transvaginal ultrasound scan (seven days after the last scan as per hospital protocol and senior review) revealed an anteverted uterus, an anterior intramural fibroid, a normal right and left ovary, and a left tubal ectopic pregnancy, with a mild amount of pelvic free fluid. In detail, Figure 1 shows the cervix, a mild amount of free fluid in the abdomen and an intramural fibroid with shadowing. Figure 2 demonstrates the size of the anterior intramural fibroid. In Figure 3, the right ovary and the dominant follicle are visible. Figure 4 presents a grossly normal left ovary, and Figure 5 demonstrates a possible left adnexal ectopic pregnancy with vascularity (Figure 6).

Recommendations

The patient was initially offered the following management options as per local hospital guidelines and individual holistic approach: 1) conservative management with repeat β-HCG monitoring and safety net advice, 2) medical management with methotrexate (an option that is controversial and a relevant contraindication due to the β-HCG levels), 3) manual vacuum aspiration (MVA) to obtain tissue samples for histology and conclude if there is intrauterine pregnancy tissue present or not, and 4) diagnostic +/- therapeutic (if an ectopic pregnancy was evident during surgery) laparoscopy along with uterine evacuation. Due to a history of keloid scarring, the patient was reluctant to undergo laparoscopy and opted for conservative management with close monitoring of β-HCG levels.

Repeat β-HCG testing 48 hours later showed a 25,710 kU/L level. The patient was reviewed by a consultant gynaecologist following the repeatedly increasing levels of β-HCG, and further management options were discussed. Given the continued rise in β-HCG levels, conservative management was not advised at this stage. The methotrexate treatment option was discussed, with thorough counselling regarding the risks of medication toxicity, treatment failure, potential ectopic rupture, and the interpregnancy interval. This option fell outside the current hospital guidelines. Surgical options, including laparoscopy with salpingectomy or salpingostomy, were also rediscussed in detail, and the associated surgical risks were explained.

The patient once again expressed a preference to avoid surgical intervention due to the risk of keloid formation. The risks of developing intrauterine adhesions from uterine evacuation were also discussed. The patient opted for another β-HCG test, which was 27,387 kU/L, taken 24 hours after her previous test. A multidisciplinary team meeting, including senior gynaecologists and plastic surgeons, was held, and a literature search on methotrexate use in high β-HCG ectopic pregnancies revealed limited data.

After a detailed discussion of risks and benefits, the patient ultimately decided to proceed with methotrexate treatment for her ectopic pregnancy - methotrexate was given intramuscularly as a single dose calculated from the patient body surface area (50 mg/m^2^). The required dose of methotrexate for the patient was 100 mg - a single dose. On day 4 post-methotrexate, her β-HCG level was 23,606 kU/L; on day 5, it decreased to 18,572 kU/L. The β-HCG levels continued to drop significantly over the next 40 days, and the patient was discharged from the early pregnancy assessment unit on day 41 post-treatment with a β-HCG level of less than 5 kU/L.

## Discussion

Ectopic pregnancy is a potentially life-threatening condition that requires prompt intervention to prevent complications. Medical management with methotrexate is a recognized alternative to surgery for unruptured ectopic pregnancies, particularly in patients with stable vital signs and low β-HCG levels. Methotrexate inhibits folate-dependent cell division, effectively terminating the pregnancy [3].

Methotrexate is most effective when β-HCG levels are below 5,000 kU/L [4]. As β-HCG levels increase, treatment success decreases; studies show that methotrexate is less effective in patients with β-HCG levels over 10,000 kU/L [5]. In such cases, surgical intervention, typically through laparoscopy, is often recommended to minimise the risk of tubal rupture and severe haemorrhage [6].

Nevertheless, methotrexate can be successful in some patients with higher β-HCG levels. Barnhart et al. found that though higher β-HCG levels correlate with increased treatment failure, methotrexate remains an option for patients who wish to avoid surgery [7]. A study by Lipscomb et al. demonstrated that while success rates decline with rising β-HCG levels, methotrexate can still be effective with careful monitoring in some patients [8].

Factors influencing methotrexate success in high β-HCG ectopic pregnancies include the size of the ectopic mass, the presence or absence of fetal cardiac activity, and the patient’s clinical stability. Smaller ectopic masses and the absence of cardiac activity are associated with higher success rates, even in patients with elevated β-HCG levels. The absence of a significant amount of free fluid in the pelvis, as seen in this case, is also a favourable prognostic indicator [6-8].

This case highlights the importance of individualized care and shared decision-making. The patient preferred to avoid surgery due to a history of keloid scarring. After extensive counselling about the risks and benefits, she chose to proceed with methotrexate despite elevated β-HCG levels. This decision was made in close collaboration with the healthcare team, which involved senior gynaecologists and plastic surgeons.

The involvement of a multidisciplinary team (MDT) was crucial in this case. The presence of plastic surgeons allowed for a detailed discussion of the risks associated with surgical scarring and potential keloid formation, which was a significant concern for the patient. This collaboration underscored the importance of tailoring management strategies to address both medical and psychosocial factors influencing patient care.

The successful outcome of this case underscores the potential for methotrexate in carefully selected high β-HCG ectopic pregnancies, particularly when patients are closely monitored and fully informed. A systematic review by Hajenius et al. emphasised the importance of patient selection and monitoring in achieving successful outcomes with methotrexate in high β-HCG cases [4].

While surgical management remains the standard for high β-HCG ectopic pregnancies, this case illustrates that methotrexate can be a viable option for some patients when prompt surgical intervention is accessible if needed. A multidisciplinary approach involving consultation with specialists is essential to address the patient’s care needs comprehensively [8].

This collaborative approach not only enhances patient safety but also empowers patients by involving them in decisions about their own care, thus promoting adherence to treatment and improving overall patient satisfaction. This case contributes to the growing body of evidence suggesting that methotrexate may be an underutilised option for high β-HCG ectopic pregnancies in selected cases. Further research and larger studies are warranted to establish more definitive guidelines for its use in this context, which could broaden treatment options for patients who are unable or unwilling to undergo surgery.

## Conclusions

This case report demonstrates the successful use of methotrexate in managing a high-β-HCG level ectopic pregnancy, emphasizing the importance of individualized care and shared decision-making. Although methotrexate is generally less effective in cases with elevated β-HCG levels, this case highlights that, with careful patient selection, thorough counselling, and close monitoring, it can still be a viable treatment option in selected cases. This case underscores the value of considering patient preferences and employing a multidisciplinary approach in managing complex clinical situations.
